# Astragalus mongholicus powder, a traditional Chinese medicine formula ameliorate type 2 diabetes by regulating adipoinsular axis in diabetic mice

**DOI:** 10.3389/fphar.2022.973927

**Published:** 2022-08-15

**Authors:** Siyuan Xu, Bixian Ye, Jinlei Li, Yonghui Dou, Yuying Yu, Yifan Feng, Lexun Wang, David Chi-Cheong Wan, Xianglu Rong

**Affiliations:** ^1^ Key Laboratory of Glucolipid Metabolic Disorder, Guangdong TCM Key Laboratory for Metabolic Diseases, Guangdong Metabolic Diseases Research Center of Integrated Chinese and Western Medicine, Ministry of Education of China, Institute of Chinese Medicine, Guangdong Pharmaceutical University, Guangzhou, China; ^2^ Department of Nursing, Medical College of Jiaying University, Meizhou, China; ^3^ School of Chinese Meteria Medica, Guangzhou University of Chinese Medicine, Guangzhou, China; ^4^ School of Biomedical Sciences, The Chinese University of Hong Kong, Hong Kong, China

**Keywords:** *Astragalus mongholicus* powder, type 2 diabetes mellitus, insulin resistance, adipoinsular axis, leptin

## Abstract

The global morbidity of obesity and type 2 diabetes mellitus (T2DM) has dramatically increased. Insulin resistance is the most important pathogenesis and therapeutic target of T2DM. The traditional Chinese medicine formula Astragalus mongholicus powder (APF), consists of *Astragalus mongholicus* Bunge [Fabaceae], *Pueraria montana* (Lour.) Merr. [Fabaceae], and *Morus alba* L. [Moraceae] has a long history to be used to treat diabetes in ancient China. This work aims to investigate the effects of APF on diabetic mice and its underlying mechanism. Diabetic mice were induced by High-fat-diet (HFD) and streptozotocin (STZ). The body weight of mice and their plasma levels of glucose, insulin, leptin and lipids were examined. Reverse transcription-polymerase chain reaction, histology, and Western blot analysis were performed to validate the effects of APF on diabetic mice and investigate the underlying mechanism. APF reduced hyperglycemia, hyperinsulinemia, and hyerleptinemia and attenuate the progression of obesity and non-alcoholic fatty liver disease (NAFLD). However, these effects disappeared in leptin deficient ob/ob diabetic mice and STZ-induced insulin deficient type 1 diabetic mice. Destruction of either these hormones would abolish the therapeutic effects of APF. In addition, APF inhibited the protein expression of PTP1B suppressing insulin–leptin sensitivity, the gluconeogenic gene PEPCK, and the adipogenic gene FAS. Therefore, insulin–leptin sensitivity was normalized, and the gluconeogenic and adipogenic genes were suppressed. In conclusion, APF attenuated obesity, NAFLD, and T2DM by regulating the balance of adipoinsular axis in STZ + HFD induced T2DM mice.

## Introduction

Nutritional excess increases the risk for obesity (Hale et al., 2015), which has developed in more than 370 million people suffering from type 2 diabetes mellitus (T2DM) (Kahn et al., 2014). T2DM has series of complications, such as dyslipidemia, non-alcoholic fatty liver disease (NAFLD), and cardiovascular disease (Cornier et al., 2008). All these metabolic disorders were introduced as glucolipid metabolic disease (GLMD) (Guo, 2017). Insulin resistance is an crucial etiology of GLMD. Leptin, a 16 kDa protein secreted by adipocyte and the key cytokine linked with obesity and T2DM (Halaas et al., 1995; Moon et al., 2013), acts as an anorectic hormone that restricts lipid storage and body weight gain (Lee et al., 2002), inhibits ectopic lipid accumulation, attenuates lipotoxicity, and improves insulin sensitivity (Minokoshi et al., 2002). This hormone has definite actions in insulin sensitivity and glucose homeostasis, although its anorectic effect contributing to insulin sensitivity is still controversial (Pelleymounter et al., 1995; Hedbacker et al., 2010).

The insulin resistance and hyperglycemia of leptin-deficient ob/ob mice can be reversed by exogenous leptin treatment (Schwartz et al., 1996; Murphy et al., 1997). But most patients with T2DM and obesity present hyperleptinemia because the leptin levels are proportional to the body mass (Moon et al., 2011). Leptin treatment is largely ineffective to improve insulin resistance and diabetic symptoms in these obese individuals (Mittendorfer et al., 2011). The poor biological function of endogenous leptin is also known as leptin resistance. Insulin stimulates the production and secretion of leptin, which in turn suppresses insulin secretion and enhance insulin sensitivity in peripheral tissues (Seufert et al., 1999; Kieffer and Habener, 2000; Alemzadeh and Tushaus., 2004; Covey et al., 2006). This leptin–insulin interaction is termed as “adipoinsular axis” (Kieffer and Habener, 2000). Dysfunction of adipoinsular axis could be a pivotal endocrine brake for insulin sensitivity and lipid oxidation.

Traditional Chinese medicine (TCM) and theory have specific merit on treating insulin resistance and GLMD (Xie and Du, 2011; Zhao et al., 2012; Guo, 2017; Gao et al., 2018). The TCM formula Astragalus mongholicus powder (APF) comprising *A. mongholicus* Bunge [Fabaceae], *P. montana* (Lour.) Merr. [Fabaceae], and *M. alba* L. [Moraceae] has been used to treat diabetes since ancient China. *A. mongholicus* and *P. montana* have potential hypoglycemic effects in diabetic animals (Hsu et al., 2003; Wang et al., 2009). In this study, the hypoglycemic effects and underlying mechanism of APF were investigated in a classic STZ + HFD induced T2DM mice, leptin deficient diabetic ob/ob mice and STZ induced T1DM mice (Kusakabe et al., 2009; Muzzin et al., 1996).

## Materials and methods

### Preparation of Astragalus mongholicus powder

Dried *A. mongholicus*, *P. montana* and *M. alba L.* were purchased from Guangzhou University of Chinese Medicine Pharmacy Co., Ltd. *A. mongholicus, P. montana*, and *Morus alba L.* were powdered and mixed in a ratio of 1:2:1. The mixture was extracted with 60% ethanol (1:8, wt/wt) for 2 h. Extraction of filtered residue was repeated for three times. The extracting solution was mixed, filtered, and concentrated to 0.12 g/ml. The concentrated solution was purified through PIPO-OO, HPD-500 macroporous resin and 732 ion exchange resin. The APF powder was obtained after freeze-drying. The ingredients of APF were determined by high-performance liquid chromatography (HPLC) and HPLC evaporation light scattering detection (HPLC-ELSD). Three representative compounds including Astragaloside IV, pueparin and calycosin-o-β-D-glucopyranoside were identified and determined as quality control. The specific content and chromatographic condition were provided in previous Supplementary Data and Table 1.

**TABLE 1:** Composition of APF.

Composition	Concentration
Astragaloside IV	5.71 mg/g
Pueparin	234.9 mg/g
Calycosin-o-β-D-glucopyranoside	0.903 mg/g

### Mice and nutrients

Normal chow, high-fat-diet (60% fat) and 8-week-old male C57BL/6J mice were purchased from Guangdong Medical Laboratory Animal Center (Foshan, China), and 8-week-old male B6.V-LepOb/LepOb (ob/ob) mice were obtained from Nanjing Biomedical Research Institution of Nanjing University (Nanjing, China). The mice were housed under pathogen-free conditions in a temperature-controlled room illuminated for 12 h every day and received humane care in accordance with the study guidelines established by the Guangzhou University of Chinese Medicine Laboratory Animal Holding Care. Following acclimation for 1 week, all C57BL/6J mice [except for 10 C57BL/6J mice as normal control (*n* = 10)] intraperitoneally received 120 mg/kg STZ once. After 3 weeks, the hyperglycemic mice were classified into four groups, namely, type 2 diabetic (*n* = 10), metformin (*n* = 10), 0.5 g/kg APF (*n* = 10), and 1.0 g/kg APF groups. The normal control group (NC) mice were fed with normal chow and treated with 5% acacia gum solution (p.o.). The T2DM, metformin, 0.5 g/kg APF, and 1.0 g/kg APF groups were fed with 60% HFD and treated with 5% acacia gum solution, 250 mg/kg metformin, 0.5 g/kg APF, and 1.0 g/kg APF (p.o.), respectively. After 12 weeks, all mice were sacrificed through cervical dislocation after anesthesia. Tissues were snap-frozen or fixed in formalin.

Type 1 diabetes (T1DM) was induced in mice by STZ. Except for 10 C57BL/6J mice as normal control (*n* = 10), all C57BL/6J mice intraperitoneally received five consecutive doses of 45 mg/kg STZ. After 2 weeks, the hyperglycemic mice were classified into T1DM (*n* = 6) and APF groups (*n* = 6) both fed with normal chow. Normal control (NC) and T1DM mice were treated with 5% acacia gum solution (p.o.). The APF group was treated with 1.0 g/kg APF (p.o.). After 6 weeks, all mice were sacrificed through cervical dislocation after anesthesia. Tissues were snap-frozen or fixed in formalin.

Ob/ob mice were designed into ob/ob (*n* = 8) and APF groups (*n* = 16) both fed with normal chow and individually treated with 5% acacia gum solution and 1.0 g/kg APF (p.o.), respectively. After 13 weeks, the APF mice were allocated to APF (*n* = 8) and APF + leptin groups (*n* = 8) that intraperitoneally received saline and 0.8 mg/kg recombinant rodent leptin, respectively, every 6:00 p.m. After 3 weeks, all mice were sacrificed through cervical dislocation after anesthesia. Tissues were snap-frozen or fixed in formalin.

All animal experimental protocols were approved by the Institutional Animal Ethics Committee of Guangdong Pharmaceutical University (GDPULACSPF No. 2012062) in compliance with the revised Animals (Scientific Procedures) Act 1986 in the UK.

### Biochemical assays

Blood sample was collected from retinal vein plexus after the mice were fasted overnight or with feeding at 9:00 a.m. Mice were anesthetized by ether. Plasma was harvested after centrifugation. Plasma glucose (Glu), triglyceride (TG), total cholesterol (TC), and low-density lipoprotein cholesterol (LDL-C) were determined using commercial kits from Rsbio (Shanghai, China). Non-esterified fatty acid (NEFA) was analyzed using NEFA assay kit from Wako (Osaka, Japan). Plasma insulin and leptin were examined using ELISA commercial kits from Cusabio (Wuhan, China). Hepatic TG and TC were extracted by isopropanol (1 mg tissue/20 μl isopropanol) allowed to stand at 4°C overnight after homogenization. Supernatants were harvested after centrifugation and determined using commercial kits.

### Oral glucose tolerance test and insulin tolerance test

Glucose (2 g/kg) was intragastrically administered to mice that fasted overnight. Insulin (1 U/kg) was intraperitoneal injected to mice that fasted for 6 h. Insulin sensitivity was evaluated using the homeostatic model assessment of insulin resistance [HOMA-IR, fasting blood glucose (mmol/L) × fasting serum insulin [(mIU/L)/22.5].

### Histology

Livers, pancreas, and adipose tissues were fixed in formalin, paraffin-embedded, sectioned, and stained with hematoxylin and eosin. For Oil Red O staining, hepatic tissues were embedded in optimal cutting temperature compound, sectioned, and stained with Oil Red O.

### Real-time polymerase chain reaction

Total RNA was isolated by homogenizing tissues in Tiangen TRIzol reagent (Beijing, China), and single standard cDNA was synthesized by using Tiangen cDNA kit (Beijing, China). Quantitative real-time PCR was performed with Thermo Scientific PikoReal 96 Real-Time polymerase chain reaction (PCR) System (Waltham, MA, United States). Primer sequences are listed in Table 2.

**TABLE 2:** Primer sequences for real-time PCR assays.

Gene	Primer
18s	F CGG​CTA​CCA​CAT​CCA​AGG​A
	R CCA​ATT​ACA​GGG​CCT​CGA​AA
GAPDH	F TGT​GTC​CGT​CGT​GGA​TCT​GA
	R TTG​CTG​TTG​AAG​TCG​CAG​GAG
FAS	F ACA​TGG​ACA​AGA​ACC​ATT​ATG​CTG​A
	R CTG​GTT​TGC​ACT​TGC​ACT​TGG​TA
ACC	F AGC​GAC​ATG​AAC​ACC​GTA​CTG​AA
	R TAG​GGT​CCC​GGC​CAC​ATA​AC
SCD1	F ATG​TCT​GAC​CTG​AAA​GCC​GAG​AA
	R GAG​CAC​CAG​AGT​GTA​TCG​CAA​GAA
HSL	F TCC​TGG​AAC​TAA​GTG​GAC​GCA​AG
	R CAG​ACA​CAC​TCC​TGC​GCA​TAG​AC
PEPCK	F TCT​TTG​GTG​GCC​GTA​GAC​CTG
	R GCC​AGG​TAT​TTG​CCG​AAG​TTG​TAG
G6Pc	F CAG​CAA​CAG​CTC​CGT​GCC​TA
	R TCC​CAA​CCA​CAA​GAT​GAC​GTT​C
PTP1B	F GAG​CAG​GAG​GGT​GTG​AAG​AG
	R CTA​GAA​GGT​CGT​GGG​CAG​AA
TCPTP	F CAA​GGC​TCA​GGC​TCA​TTG​TG
	R CCG​CCA​TAG​TCA​GTG​AAG​CA

Sequences: 5′ to 3′. Forward primers are designated by f and reverse primers by r.

### Western blot

Total protein extracts were fractionated by sodium dodecyl sulfate polyacrylamide gel electrophoresis and transferred to polyvinylidene difluoride membranes. The membranes were then blocked with 5% nonfat milk in Tris-buffered saline with Tween-20 for 2 h at room temperature and incubated with anti-PTP1B (Abcam, United States), anti-TCPTP, and anti-GAPDH (Cell Signaling Technology, United States) at 4°C overnight, rinsed three times with TBST, and incubated with respective secondary antibodies for 2 h at room temperature. Protein bands were visualized with Thermo Fisher Scientific SuperSignal West Femto Maximum Sensitivity Substrate (Rockford, United States) and captured using an Image Quant LAS4000 imaging system (Shanghai, China).

### Data analysis

All results were expressed as means ± standard error of the mean. Data were analyzed by Kolmogorov-Smirnov and Mann-Whitney U test for normal distribution analysis. Data from more than two groups were analyzed by one-way ANOVA. Student’s t test was performed to identify differences between two groups. *p < 0.05* was considered significant.

## Results

### Astragalus mongholicus powder improved insulin sensitivity in STZ + HFD induced type 2 diabetic mice

**FIGURE 1:**
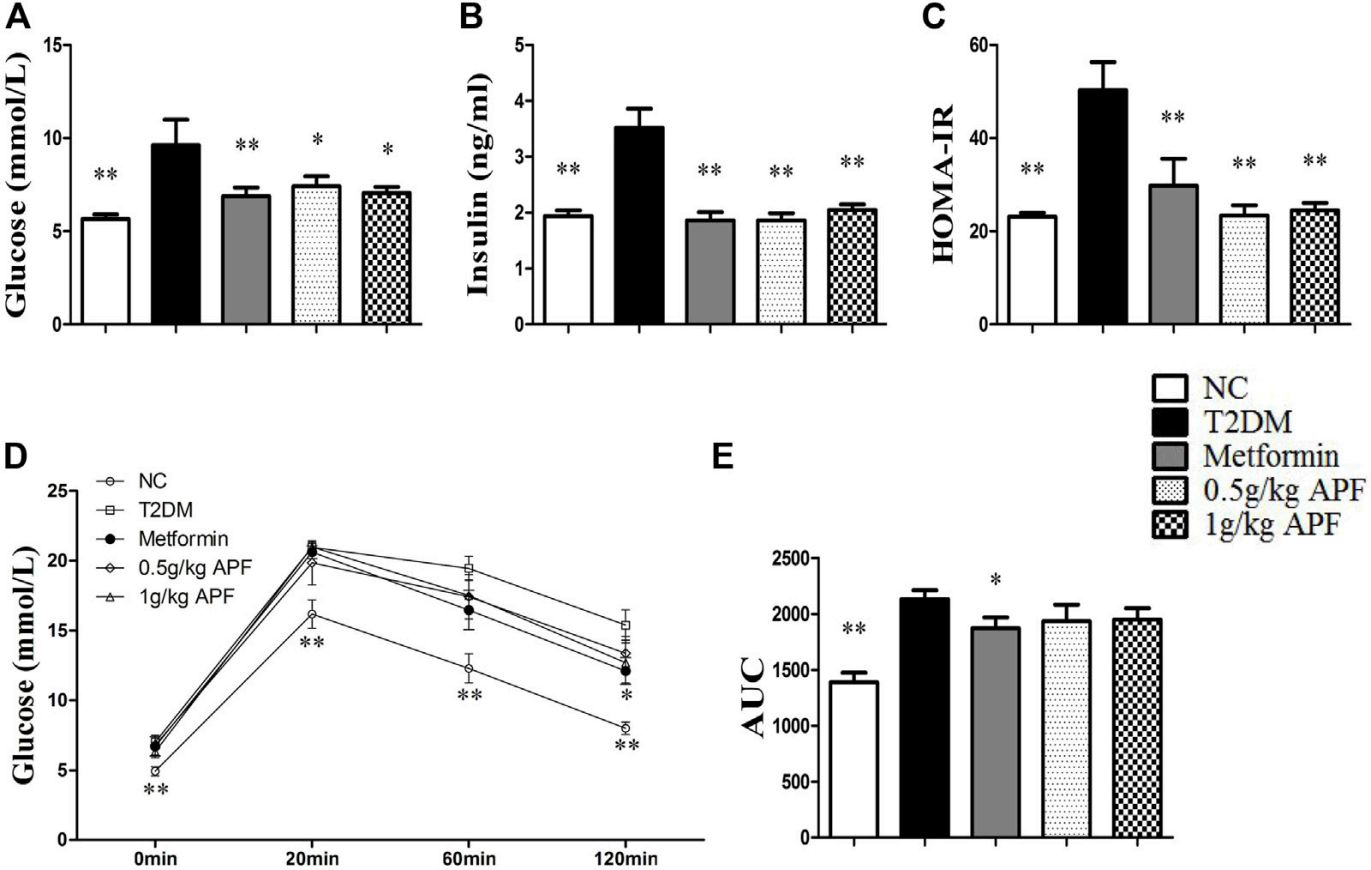
Effects of APF on insulin resistance in T2DM mice. Fasting plasma glucose **(A)**, insulin **(B)**, homeostatic model assessment of insulin resistance **(C)**, plasma glucose kinetics **(D)** and area under the curve of kinetics profiles **(E)** after 2 g/kg oral glucose administration of normal control (white bars), type 2 diabetes group (black bars), metformin group (gray bars), 0.5 g/kg APF group (spot bars) and 1 g/kg APF group (grid bars) after treatment with APF 10 weeks. Values are means ± SEMs, *n* = 8–10 per group. *p < 0.05, **p < 0.01 versus type 2 diabetes group.

After feeding with HFD 10 weeks, the T2DM group exhibited hyperglycemia, hyperlipidemia, hyperinsulinemia, impaired glucose tolerance, and elevated HOMA-IR index (Figures 1A–E; Supplementary Figures S1A,B), indicating the strong development of T2DM. However, the levels of fasting glucose, insulin, and HOMA-IR were normalized by treating with 0.5 g/kg APF, 1 g/kg APF, and metformin, respectively (Figures 1A–C). Meanwhile, STZ administration elicited the injury of morphology of pancreas in T2DM group (Supplementary Figure S1C), which was attenuated by 0.5 and 1 g/kg APF treatment (Supplemental Figure S1C). These data suggested that APF reshapes the STZ-injured pancreas and reduces the compensatory secretion of insulin. Therefore, the HFD-induced insulin resistance and hyperinsulinemia are alleviated by APF treatment.

### Astragalus mongholicus powder ameliorated obesity and non-alcoholic fatty liver disease development

**FIGURE 2:**
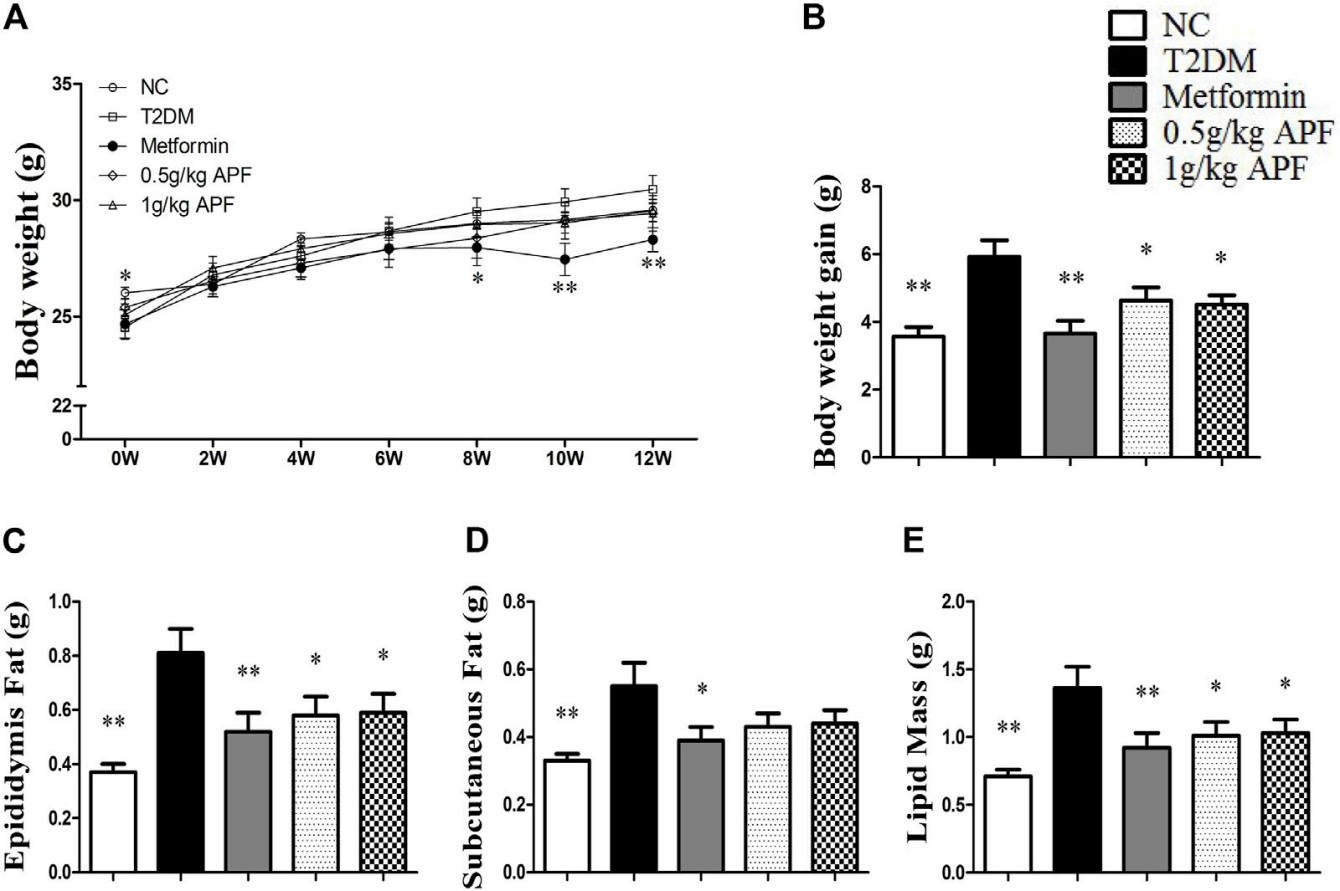
Effects of APF on obesity in T2DM mice. Body weight changes **(A)**, body weight gain **(B)**, epidydymal fat mass **(C)**, subcutaneous fat mass **(D)** and total fat mass **(E)** of normal control (white bars), type 2 diabetes group (black bars), metformin group (gray bars), 0.5 g/kg APF group (spot bars) and 1 g/kg APF group (grid bars) after treatment with APF 12 weeks. Values are means ± SEMs, *n* = 8–10 per group. *p < 0.05, **p < 0.01 versus type 2 diabetes group.

**FIGURE 3:**
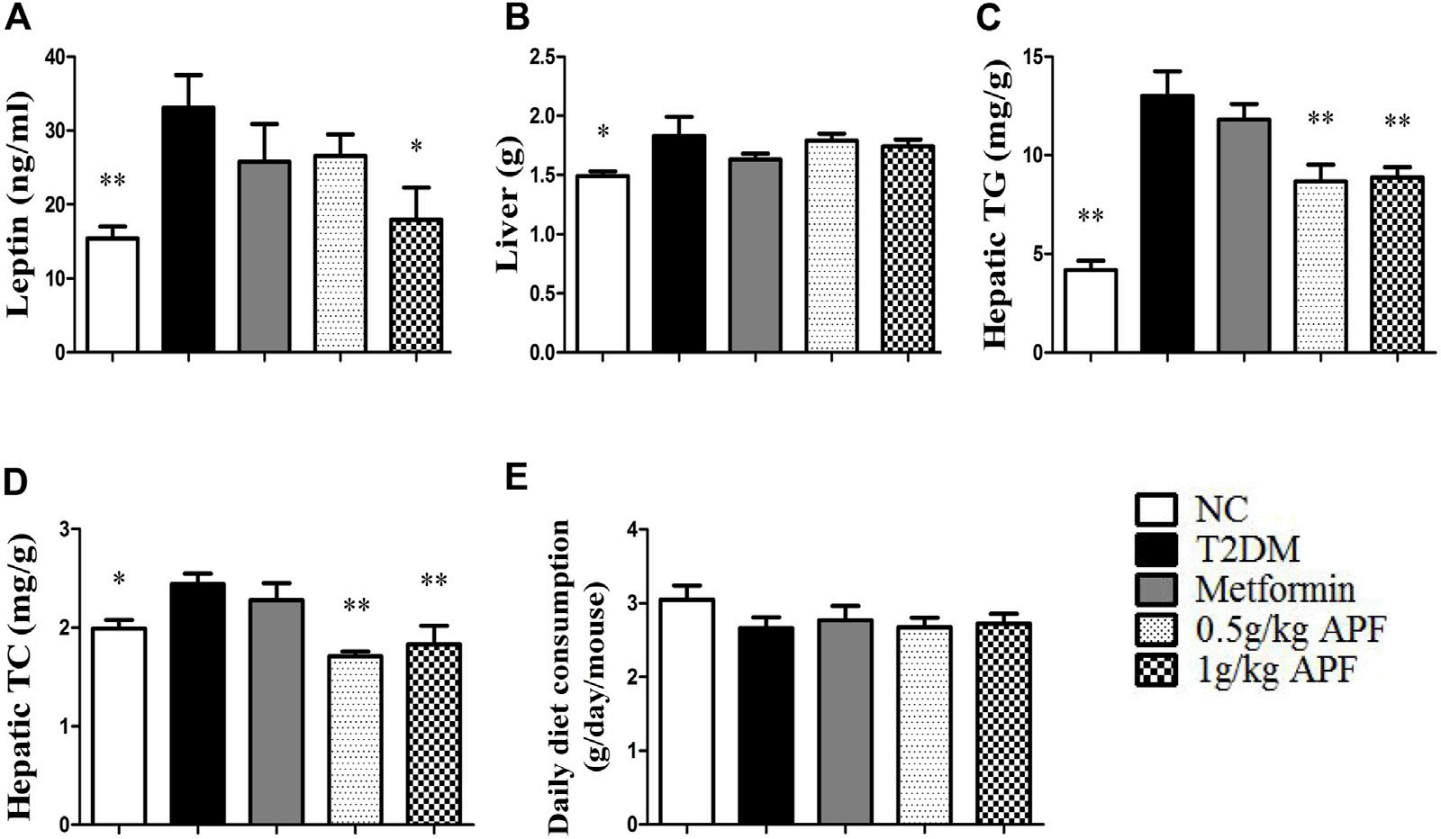
Effects of APF on hepatic steatosis in T2DM mice. Plasma leptin **(A)**, liver weight **(B)**, hepatic TG **(C)**, hepatic TC **(D)** and daily diet consumption **(E)** of normal control (white bars), type 2 diabetes group (black bars), metformin group (gray bars), 0.5 g/kg APF group (spot bars) and 1 g/kg APF group (grid bars) after treatment with APF 12 weeks. Values are means ± SEMs, *n* = 8–10 per group. *p < 0.05, **p < 0.01 versus type 2 diabetes group.

STZ administration significantly reduced the body weight of mice (Figure 2A). After feeding with HFD, the T2DM group had higher body weight gain than the NC group (Figure 2B). Fat mass weight was also monitored, and the results showed that epididymal fat, subcutaneous fat, and total lipid mass were significantly enhanced after HFD feeding (Figures 2C–E). Plasma leptin levels proportional to the fat mass also increased with HFD feeding (Figure 3A). In addition to body mass, liver weight, and hepatic TC, the TG content was significantly higher than that in the NC group (Figures 3B–D). Histological staining also displayed substantial amounts of hepatic macrovesicular steatosis in the T2DM group (Supplementary Figure S2). These results showed that the T2DM group turned obese and developed NAFLD accompanied with insulin resistance. With regard to the biological function of leptin in anti-ectopic lipid accumulation, the T2DM group showed insulin and leptin resistance.

APF treatment significantly inhibited the increase in body weight gain (Figure 2B). Epididymal fat mass and total lipid mass also deceased with APF intervention (Figures 2C,E). Moreover, 1 g/kg APF treatment diminished the diameter of the adipocyte and might have contributed to improving the endocrine function of adipose tissues (Supplementary Figure S3). Thus, the hyperleptinemia of T2DM mice was normalized by 1 g/kg APF treatment (Figure 3A). Finally, APF administration reduced the hepatic TC and TG contents and remarkably diminished the hepatic macrovesicular steatosis without effects in daily diet consumption (Figures 3C–E; Supplementary Figure S2). The results indicated that APF attenuates obesity, hyperleptinemia and NAFLD. Based on the effects of APF in insulin sensitivity. We considered APF reversed the insulin-leptin resistance though regulating the adipoinsular axis.

### Astragalus mongholicus powder failed to ameliorate insulin resistance, obesity and non-alcoholic fatty liver disease in leptin-deficient ob/ob mice

**FIGURE 4:**
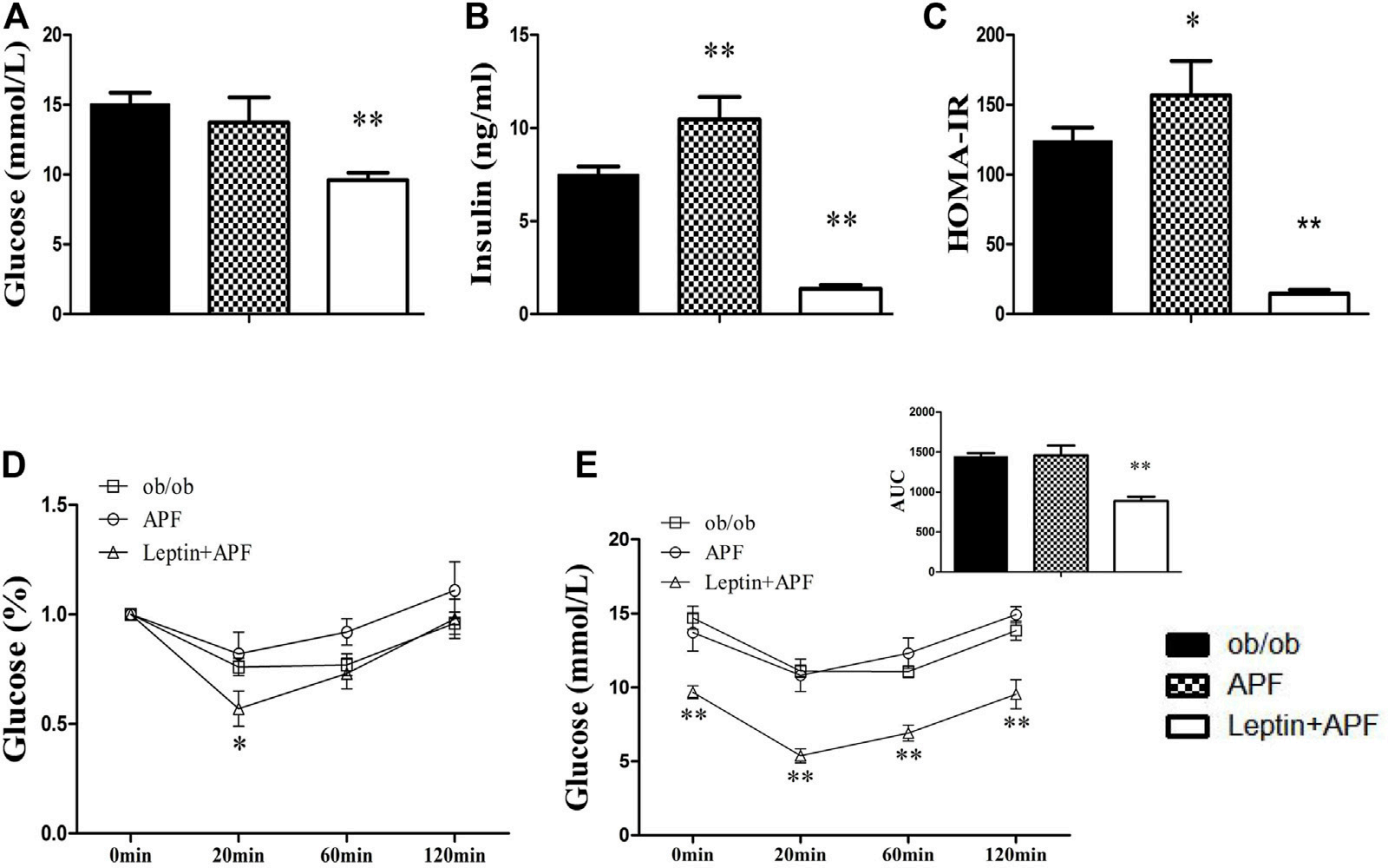
Effects of APF and supplementary Leptin on insulin resistance in ob/ob mice. Fasting plasma glucose **(A)**, insulin **(B)**, homeostatic model assessment of insulin resistance **(C)**, intraperitoneal insulin tolerance test **(D)** and area under the curve of kinetics profiles **(E)** after 1 U/kg intraperitoneal injected insulin administration of ob/ob group (black bars), APF group (grid bars) and APF + leptin group (white bars) after treatment with APF 15 weeks or APF + lepin 2 weeks. Values are means ± SEMs, *n* = 6-8 per group. *p < 0.05, **p < 0.01 versus ob/ob group.

Leptin-deficient ob/ob mice were selected to validate whether leptin is essential for APF improve insulin sensitivity by regulating adipoinsular axis (Supplementary Figure S4C). In the status of absent of leptin, APF could not normalize the hyperglycemia and hyperinsulinemia of ob/ob mice (Figures 4A,B). APF even increased the levels of plasma insulin and HOMA-IR index (Figures 4B,C). We considered that APF may still influence the endocrine function of pancreas, but the effects are unpredictable without leptin. Monitoring revealed that insulin resistance was robust in ob/ob mice with APF treatment (Figures 4D,E).

**FIGURE 5:**
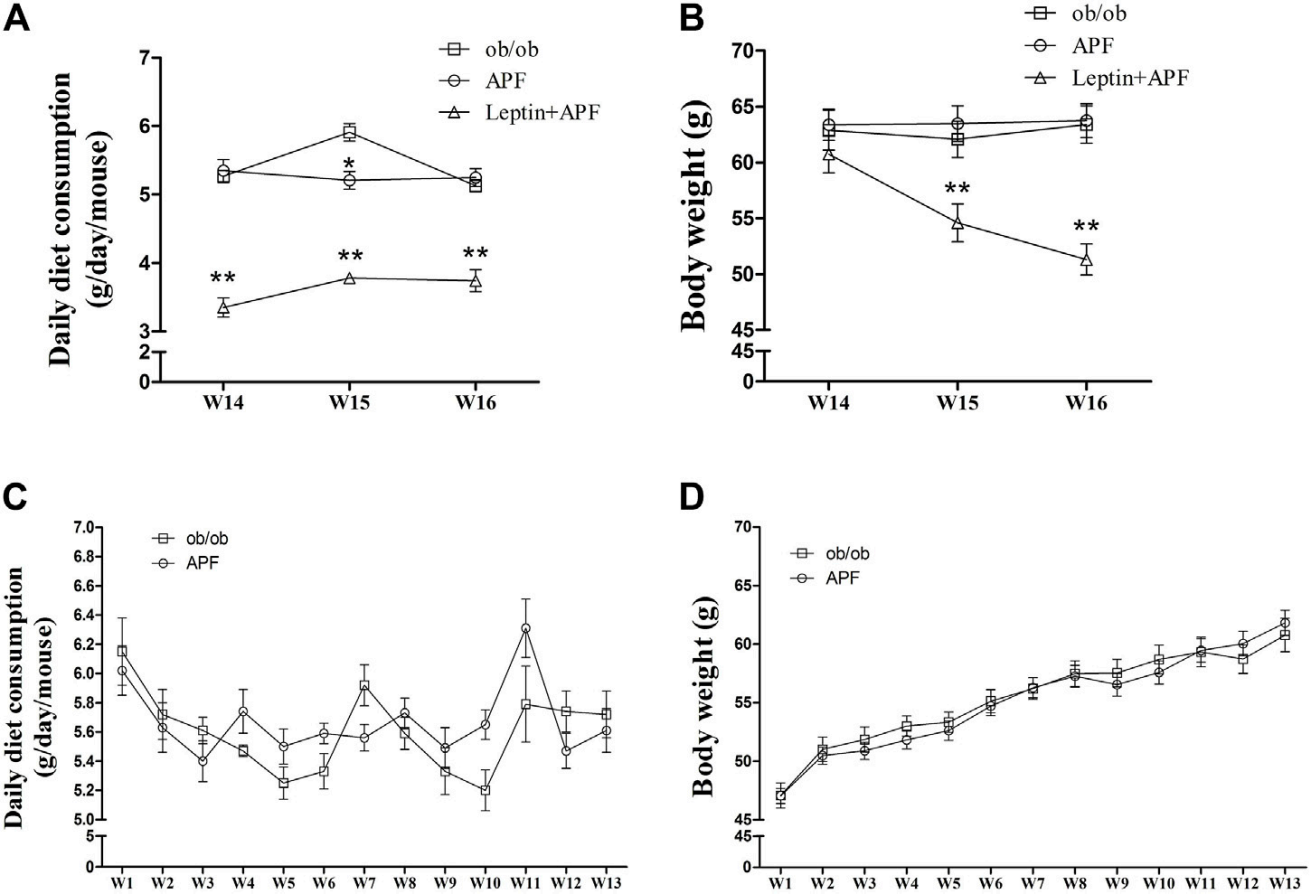
Effects of APF and supplementary Leptin on ob/ob mice. Daily diet consumption **(A,C)**, body weight changes **(B,D)** of ob/ob group (white squares), APF group (white cycles) and APF + leptin group (white trigons) after treatment with APF 16 weeks or APF + letpin 3 weeks. Values are means ± SEMs, *n* = 7–8 per group. *p < 0.05, **p < 0.01 versus ob/ob group.

**FIGURE 6:**
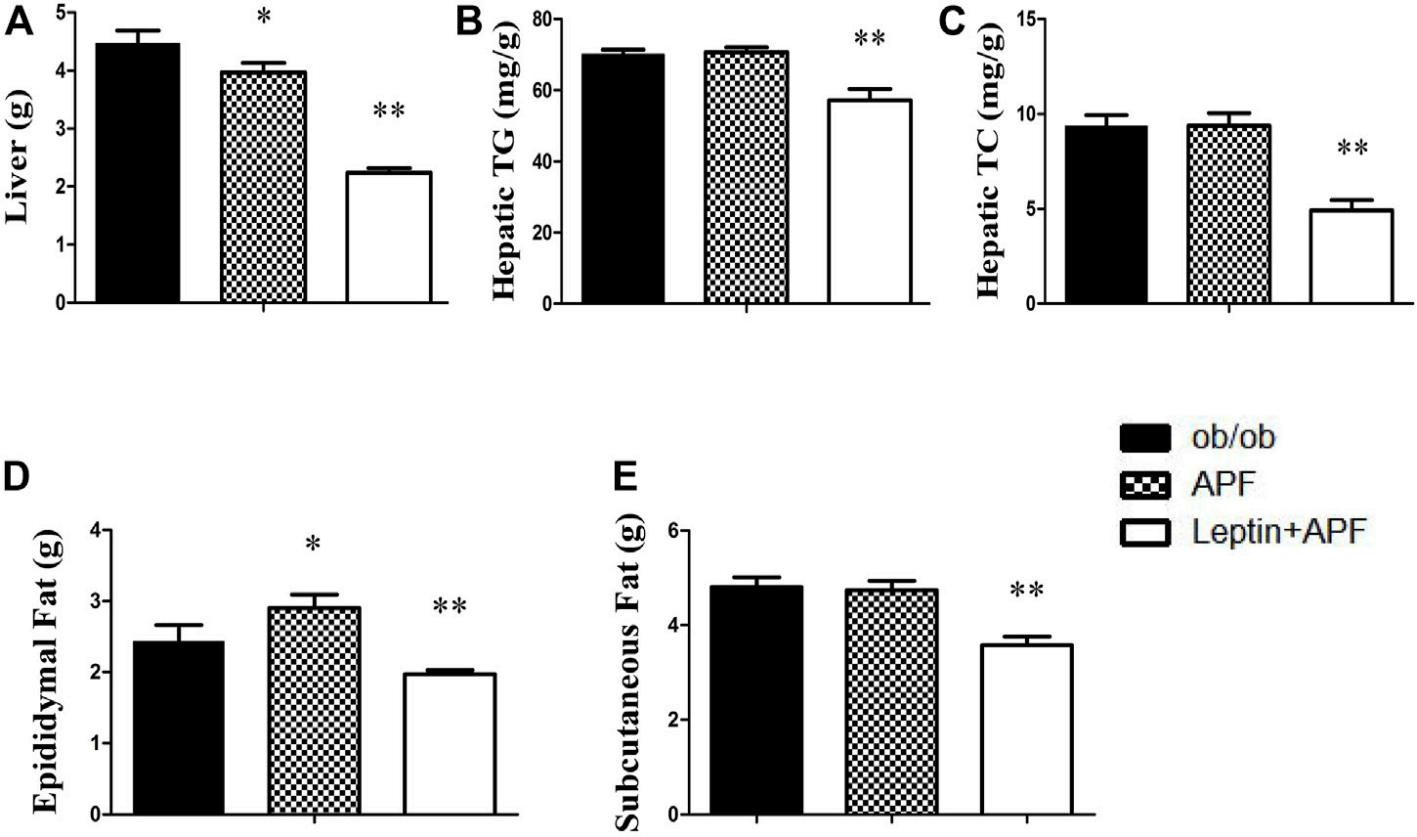
Effects of APF and supplementary Leptin on abdominal obesity in ob/ob mice. Liver weight **(A)**, hepatic TG **(B)**, hepatic TC **(C)**, epidydymal fat mass **(D)** and subcutaneous fat mass **(E)** of ob/ob group (black bars), APF group (grid bars) and APF + leptin group (white bars) after treatment with APF 16 weeks or APF + lepin 3 weeks. Values are means ± SEMs, *n* = 7–8 per group. *p < 0.05, **p < 0.01 versus ob/ob group.

Based on the physiological effects of adipoinsular axis, the pathological features of obesity and NAFLD in ob/ob mice with APF administration were examined. Different from that in STZ + HFD induced T2DM mice, the weight-loss effects of APF were not observed in ob/ob mice (Figures 5B,D). Meanwhile, the hepatic macrovesicular steatosis of ob/ob mice was not reversible by APF intervention (Supplementary Figure S5). Although APF reduced the hepatic weight, APF failed to decrease the levels of hepatic TG and TC (Figures 6A–C). On the basis of body weight, the fat mass of ob/ob mice was examined. APF could not reduce the subcutaneous fat mass and even enhanced the epididymal fat mass in ob/ob mice (Figures 6D,E). As implied by these data, the destruction of adipoinsular axis in the leptin side would abolish the effects of APF. APF lost its effects of anti-insulin resistance, obesity, and NAFLD without leptin regulation but still had minimal influence on pancreas and adipose tissues.

### Leptin supplementary treatment reversed the metabolic syndrome of ob/ob mice

Ob/ob mice were administered with recombinant rodent leptin (*i.p.*) after APF treatment for 13 weeks (Supplementary Figure S4C). Hyperglycemia, hyperinsulinemia, HOMA-IR, and insulin tolerance were normalized after leptin treatment (Figures 4A–E). Leptin supplementary treatment significantly inhibited their daily diet consumption and body weight (Figures 5A–D). For NAFLD and fat mass, leptin administration attenuated hepatic steatosis and reduced hepatic TC and TG levels (Supplementary Figure S5; Figures 6A–C). Therefore, fat mass, including epididymal and subcutaneous fat, was decreased by leptin supplement (Figures 6D,E). Leptin also diminished the diameter of ob/ob mouse adipocytes, which was the same effect as APF in STZ + HFD-induced T2DM mice (Supplementary Figures S3, S6). All these data were confirmed by previous studies of leptin and validated the effects of adipoinsular axis from another point of view.

### Astragalus mongholicus powder lost its hypoglycemic effects in insulin-deficient type 1 diabetes mice

**FIGURE 7:**
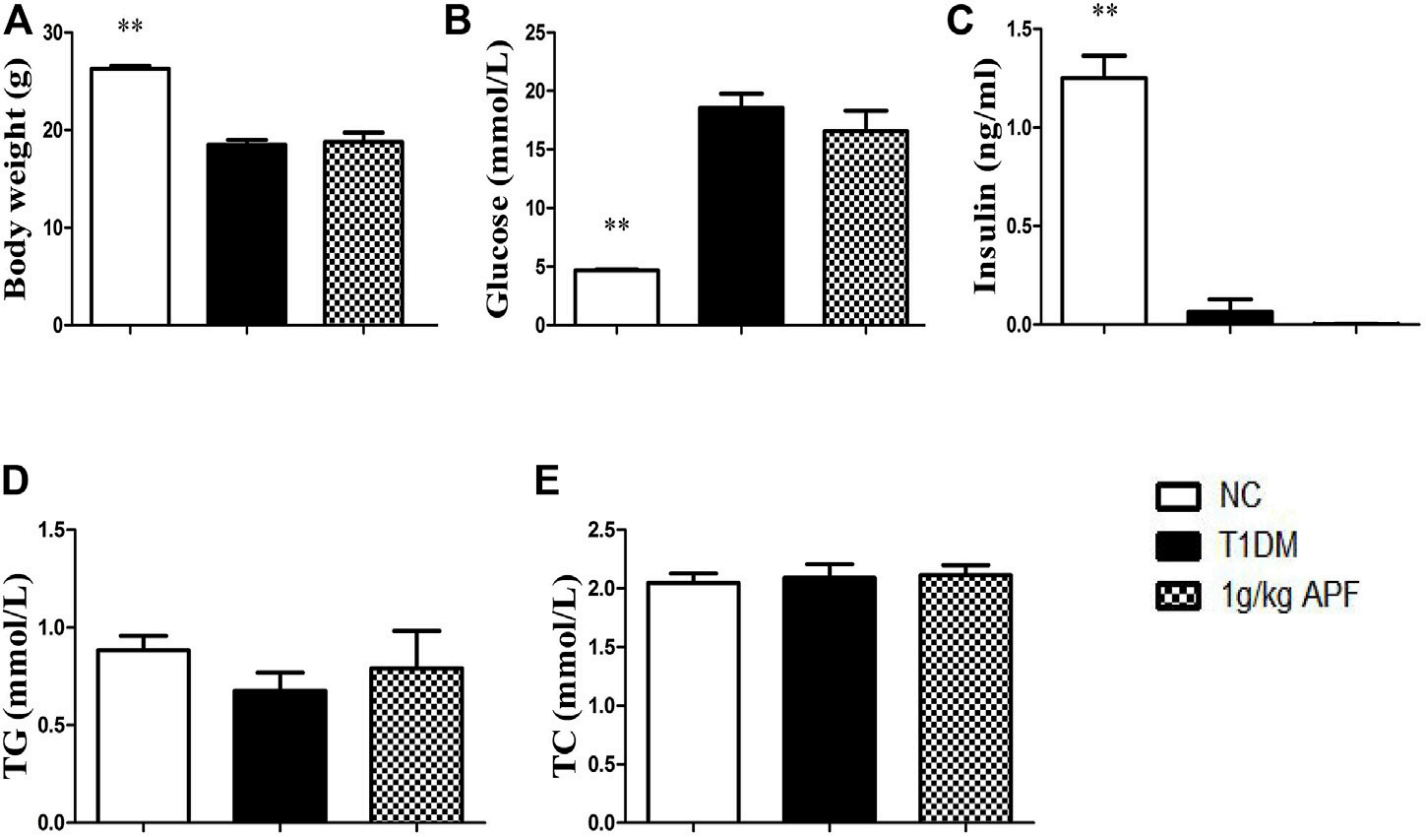
Effects of APF in T1DM mice. Body weigt **(A)**, plasma glucose **(B)**, insulin **(C)**, TG **(D)** and TC **(E)** of normal control (white bars), type 1 diabetes group (black bars) and APF group (grid bars) after treatment with APF 6 weeks. Values are means ± SEMs, *n* = 6–10 per group. *p < 0.05, **p < 0.01 versus T1DM group.

We hypothesized that APF ameliorate diabetes by regulating adipoinsular axis. Except for the leptin, insulin also must be essential for the hypoglycemic effects of APF. As expected, APF could not ameliorate diabetes and metabolic syndromes without leptin presence in ob/ob mice. Then, a classic T1DM mice model was established to investigate the anti-diabetic effects of APF in absence of inuslin. After STZ administration, the mice showed body loss, hyperglycemia, and insulin deficiency (Figures 7A–C). However, the hypoglycemic effects of APF in T2DM mice was not observed in T1DM mice (Figure 7B). In addition, the levels of plasma insulin was hardly detected with APF treatment (Figure 7C). Meanwhile, APF also had no effects in TC and TG in T1DM mice (Figure 7D, E). Therefore, both hormones (leptin and insulin) are indispensable in regulating the adipoinsular axis by APF. Abolishment of each hormone would disrupt the effects of APF for anti-diabetes, obesity, and NAFLD.

### Potential mechanism of astragalus mongholicus powder in adipoinsular axis

**FIGURE 8:**
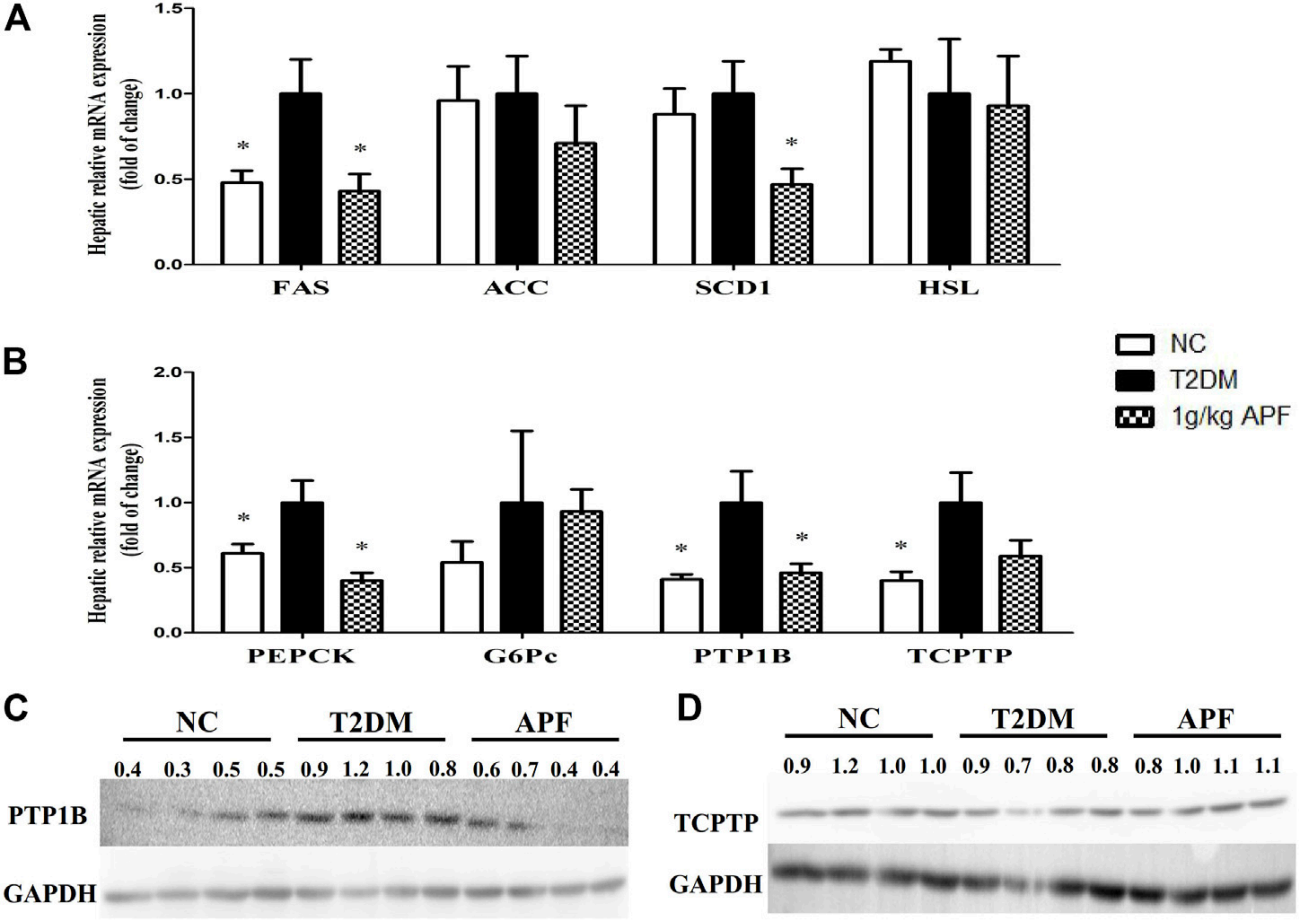
Effects of APF on liver in T2DM mice. Hepatic FAS, ACC, SCD1, HSL **(A)**, PEPCK, G6Pc, PTP1B, TCPTP **(B)** relative mRNA expression and protein expression of P1P1B **(C)** and TCPTP **(D)** of normal control (white bars), type 2 diabetes group (black bars) and 1 g/kg APF group (grid bars) after treatment with APF 12 weeks. Values are means ± SEMs, *n* = 4–5 per group. *p < 0.05, **p < 0.01 versus type 2 diabetes group.

APF ameliorated insulin resistance and NAFLD by improving the adipoinsular axis. The underlying mechanism of these effects was then investigated. Hepatic lipid metabolic gene expression was determined based on the decreased hepatic lipid accumulation by APF. APF treatment significantly suppressed the expression of lipogenic gene, fatty acid synthase (FAS), and stearoyl-CoA desaturase-1 (SCD1) (Figure 8A). Both genes contribute to hepatic *de novo* lipogenesis (Wang et al., 2015). On the contrary, APF alleviated the hepatic expression of gluconeogenesis gene phosphoenolpyruvate carboxykinase (PEPCK), a target gene for leptin to improve insulin sensitivity (Burcelin et al., 1999). These data suggested that APF improves the hepatic biological effects of insulin–leptin and suppresses the expression of hepatic lipogenic and gluconeogenic genes. Protein tyrosine phosphatase 1B (PTP1B) and T-cell protein tyrosine phosphatase (TCPTP) are negative regulators of insulin and leptin reaction (Thareja et al., 2012; Zhang et al., 2015). Thus, their expression levels were determined by real-time PCR and Western blot. The genetic and protein expression levels of PTP1B in STZ + HFD-induced T2DM mice were significantly enhanced (Figures 8B,C), and this finding was consistent with the phenotype of dysfunction of adipoinsular axis in mice. However, APF treatment significantly reversed the overexpression of PTP1B but not TCPTP (Figures 8B–D), implying that APF improves the adipoinsular axis balance by suppressing PTP1B and consequently contributes to the attenuation of obesity, NAFLD, and T2DM.

## Discussion

Overnutrition triggers lipid excess in adipose tissues and increases lipolysis and circulating lipids. The excess circulating lipid causes ectopic lipid accumulation in peripheral tissues (liver, skeletal muscles, and β cells); induces lipotoxicity, chronic inflammation, and endoplasmic reticulum stress; and contribute to obesity, NAFLD, and insulin resistance development, which means modulation the interplay between liver and adipose tissue are critical in GLMD development (Guilherme et al., 2008; Samuel and Shulman, 2012; Ye et al., 2017). Leptin plays a pivotal role in energy homeostasis and controls body weight as an anorexigenic hormone in hypothalamic area (Cowley et al., 2001; Balthasar et al., 2004). This hormone also prevents ectopic lipid accumulation and lipotoxicity by promoting lipid oxidation (Minokoshi et al., 2002; Zeng et al., 2015) and even directly inhibits the secretion of insulin and improves insulin sensitivity (Liu et al., 2003; Laubner et al., 2005). In turn, the lipogenic hormone insulin promotes adiposity and increases leptin production that is proportional to fat mass (Kim et al., 1998; Aas et al., 2009). The balance of feedback (adipoinsular axis) contributes to global energy homeostasis, and its dysfunction is a primary pathogenesis linked to obesity, NAFLD, and T2DM (Vickers et al., 2001; Simmons and Breier, 2002).

In this research, well-recognized HFD + STZ induced obese T2DM mice were established to investigated the effects of APF. After STZ broke the islet form, HFD induced hyperglycemia, hyperinsulinemia, and insulin resistance. Meanwhile, HFD promoted obesity, hepatic steatosis, and hyperleptinemia in mice. These data indicated that the mice lost adipoinsular axis balance and developed insulin–leptin resistance, which strongly contributes to T2DM and NAFLD deterioration. However, APF treatment reversed hyperleptinemia and suppressed NAFLD and obesity development. Histological staining displayed that APF could diminish the diameter of adipocytes. Hypertrophic adipocytes prompt the endocrine dysfunction of adipose tissues (Gustafson et al., 2015). Thus, APF may have potential effects on the endocrine function of adipose tissues and leptin metabolism. APF also normalizes the levels of plasma glucose, insulin, and HOMA-IR index. Moreover, the injured islets were restored with APF treatment. Hence, we considered that APF could improve the endocrine function of islet and attenuate insulin compensatory secretion and insulin resistance. A previous study reported that leptin administration enhances islet transplant performance in diabetic mice (Denroche et al., 2013). Thus, we suggested that both effects of APF on reversing hyperleptinemia and hyperinsulinemia were based on balancing the adipoinsular axis. Insulin and leptin resistance was consequently ameliorated, and obesity, T2DM, and NAFLD were repressed in mice.

Several compounds, such as peroxisome proliferators-activated receptor alpha (PPARα) agonist, can ameliorate insulin resistance and obesity and normalize hyperleptinemia and hyperinsulinemia independent of adipoinsular axis (Ye et al., 2001; Jeong and Yoon, 2009). The leptin-deficient ob/ob mice and insulin-deficient T1DM mice were established to confirm that insulin and leptin are essential for APF anti-metabolic syndromes by regulating adipoinsular axis. In ob/ob mice, obesity and hepatic steatosis were refractory to APF treatment without leptin. In addition to obesity, insulin resistance was still robust with APF treatment. APF treatment enhanced the levels of plasma insulin and epididymal fat mass in ob/ob mice. These effects were contrary to APF influences on T2DM mice. We considered that APF still had potential effects on tissues of adipoinsular axis (pancreas and adipose tissues), but these actions became unpredictable when the adipoinsular axis was abolished. The effects of APF on anti-obesity, NAFLD and T2DM were not observed in ob/ob mice. These data indicated that APF improves insulin resistance and metabolic syndrome in a leptin-dependent manner. APF had no significant effects on the daily diet consumption of ob/ob mice and T2DM mice. We considered that the effects of APF on adipoinsular axis were independent of leptin’s action on the central nervous system. The anorectic function of leptin in the central nervous system is independent of insulin sensitivity and NAFLD (Hedbacker et al., 2010). Meanwhile, the hypoglycemic effects of APF was lost in STZ induced T1DM mice that were deficient in insulin, which means the hypoglycemic effects of APF rely on insulin existing. On the basis of these data, APF ameliorates obesity, NAFLD, and diabetes by regulating adipoinsular axis. Destruction of each side of adipoinsular axis would diminish the effects of APF.

**FIGURE 9:**
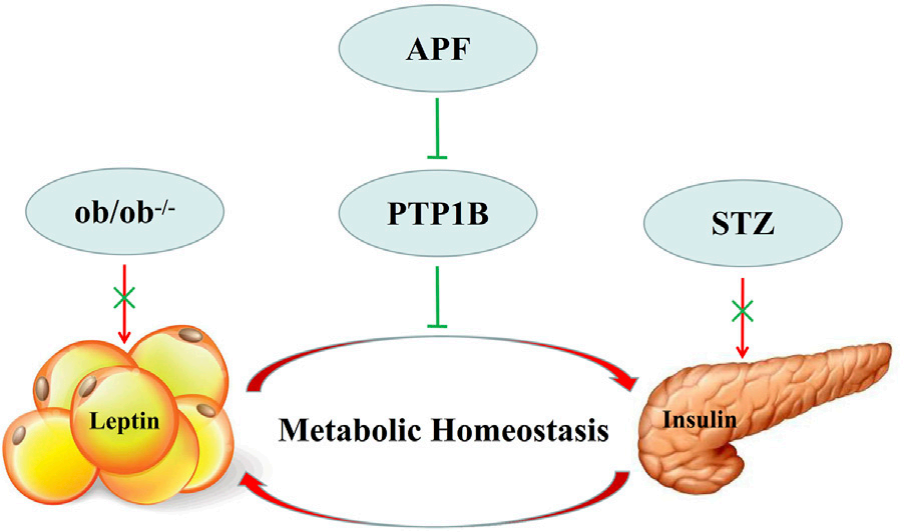
Model describing of APF in adipoinsular axis. APF suppress the inhibition effects of PTP1B in adipoinsular axis, which contribute to the balance of adipoinsular axis in metabolic homeostasis. This effect can be abolished by blocking the insulin-leptin feedback.

Although the balance of adipoinsular axis is crucial for energy homeostasis, leptin and insulin sensitivity may be suppressed by some endogenetic signailing pathways (Taniguchi et al., 2006; Balland and Cowley, 2015). PTP1B and TCPTP contribute to insulin resistance (Galic et al., 2003; Delibegovic et al., 2009). However, deletion of PTP1B could not improve insulin resistance in leptin receptor mutant db/db mice (Ali et al., 2009; Tsou et al., 2014). These data revealed that the suppression of PTP1B improves insulin sensitivity in a leptin-dependent manner. Hence, PTP1B could be a candidate therapeutic target for balancing adipoinsular axis. In this research, APF significantly decreased the hepatic mRNA and protein expression of PTP1B in T2DM mice. Therefore, the expression levels of gluconeogenesis and lipogenesis (PEPCK, FAS, and SCD1) hepatic genes were inhibited. These results were supported by leptin agonist attenuating insulin resistance and reducing the expression of gluconeogenic and lipogenic genes (Burcelin et al., 1999; Tsuchiya et al., 2012). Therefore, APF diminishes the suppressive effects of PTP1B on adipoinsular axis and consequently improves leptin and insulin sensitivity. The gluconeogenic and lipogenic genes were then repressed. The role of adipoinsular axis in energy homeostasis has been reported, and the mechanism of leptin in insulin sensitivity has been revealed (Buettner et al., 2006; Berglund et al., 2012; Könner and Brüning, 2012; Luan et al., 2014). However, the feedback mechanism of adipoinsular axis required further study. This research illustrated that APF attenuates metabolic diseases by balancing the adipoinsular axis due to its suppressive effects on PTP1B expression (Figure 9). However, the specific mechanism of APF in adipoinsular axis is still unclear. In addition to the adipoinsular axis, APF decreased the levels of plasma low density lipoprotein (LDL) in ob/ob mice (Supplementary Figure S4B). This effect may contribute to other diseases. Further works are required to investigate the mechanism of APF in the adipoinsular axis.

## Conclusion

APF regulated the balance of adipoinsular axis in STZ + HFD induced T2DM mice due to its suppressive effects on PTP1B expression. Hyperleptinemia/leptin resistance and hyperinsulinemia/insulin resistance were ameliorated. As a result, the hepatic genes of gluconeogenesis and lipogenesis were inhibited, and hyperglycemia, hepatic steatosis, and fat mass excess were attenuated. Finally, GLMD (obesity, NAFLD, and T2DM) development were repressed by APF treatment.

## Data Availability

The raw data supporting the conclusion of this article will be made available by the authors, without undue reservation.

## Supplementary material

SUPPLEMENTARY FIGURE S1 Fasting plasma TG , **(A)** TC **(B)** and representative hematoxylin and eosin staining of pancreas of normal control (white bars), type 2 diabetes group (black bars), metformin group (gray bars), 0.5 g/kg APF group (spot bars) and 1 g/kg APF group (grid bars) after treatment with APF 12 weeks. Values are means ± SEMs, *n* = 8–10 per group. **p* < 0.05, ***p* < 0.01 versus type 2 diabetes group.

SUPPLEMENTARY FIGURE S2 Representative hematoxylin and eosin staining of livers from each group after treatment with APF 12 weeks.

SUPPLEMENTARY FIGURE S3 Representative hematoxylin and eosin staining of epididymal fat from each group after treatment with APF 12 weeks.

SUPPLEMENTARY FIGURE S4 Fasting plasma TG, NEFA **(A)** TC, LDL-C **(B)** and research protocol **(C)** of ob/ob group (black bars) and APF group (grid bars) after treatment with APF 13 weeks. Values are means ± SEMs, *n* = 8–16 per group. **p* < 0.05, ***p* < 0.01 versus ob/ob group.

SUPPLEMENTARY FIGURE S5 Representative hematoxylin, eosin staining or Oil red O staining of pancreas and livers from each group after treatment with APF 16 weeks or APF+lepin 3 weeks.

SUPPLEMENTARY FIGURE S6 Representative hematoxylin and eosin staining of epididymal fat and subcutanoues fat after treatment with APF 16 weeks or APF+lepin 3 weeks.

## Abbreviations

ACC, Acetyl-CoA carboxylase; APF, Astragalus mongholicus powder; FAS, fatty acid synthase; Glu, glucose; G6Pc, Glucose-6-phosphatase, catalytic subunit; HFD, high fat diet; HOMA-IR, homeostasis model of assessment for insulin resistance; HSL, hormone sensitive lipase; ITT, insulin tolerance test; LDL-C, low-density lipoprotein cholesterol; NAFLD, non-alcoholic fatty liver disease; NC, normal control; NEFA, non-esterified fatty acid; OGTT, oral glucose tolerance test; PCR, polymerase chain reaction; PEPCK, phosphoenolpyruvate carboxykinase; PPARα, peroxisome proliferator activated receptor α; PTP1B, protein tyrosine phosphatase 1B; SCD1, stearoyl-CoA desaturase-1; STZ, streptozotocin; TC, total cholesterol; TCM, traditional Chinese medicine; TCPTP, T-cell protein tyrosine phosphatase; T1DM, type 1 diabetes mellitus; T2DM, type 2 diabetes mellitus; TG, triglyceride.
