# Supplementary material for: Astragalus mongholicus powder, a traditional Chinese medicine formula ameliorate type 2 diabetes by regulating adipoinsular axis in diabetic mice
Source: Front Pharmacol. 2022 Aug 15;13:973927. doi: 10.3389/fphar.2022.973927 (PMC9420938; doi:10.3389/fphar.2022.973927)
Supplement: Supplementary file 1 [file DataSheet2.pdf]

Figures legend

Supplemental Figure 1 Fasting plasma TG (A), TC (B) and representative hematoxylin and eosin staining of pancreas of normal control (white bars), type 2 diabetes group (black bars), metformin group (gray bars), 0.5g/kg APF group (spot bars) and 1g/kg APF group (grid bars) after treatment with APF 12 weeks. Values are means ± SEMs, n=8-10 per group. *$P<0.05$*, **$P<0.01$* versus type 2 diabetes group.

Supplemental Figure 2 Representative hematoxylin and eosin staining of livers from each group after treatment with APF 12 weeks.

Supplemental Figure 3 Representative hematoxylin and eosin staining of epididymal fat from each group after treatment with APF 12 weeks.

Supplemental Figure 4 Fasting plasma TG, NEFA (A), TC, LDL-C (B) and research protocol (C) of ob/ob group (black bars) and APF group (grid bars) after treatment with APF 13 weeks. Values are means ± SEMs, n=8-16 per group. *$P<0.05$, **$P<0.01$* versus ob/ob group.

Supplemental Figure 5 Representative hematoxylin, eosin staining or Oil red O staining of pancreas and livers from each group after treatment with APF 16 weeks or APF+lepin 3 weeks.

Supplemental Figure 6 Representative hematoxylin and eosin staining of epididymal fat and subcutanoues fat after treatment with APF 16 weeks or APF+lepin 3 weeks.
