# Supplementary figures and images for: Astragalus mongholicus powder, a traditional Chinese medicine formula ameliorate type 2 diabetes by regulating adipoinsular axis in diabetic mice
Source: Front Pharmacol. 2022 Aug 15;13:973927. doi: 10.3389/fphar.2022.973927 (PMC9420938; doi:10.3389/fphar.2022.973927)

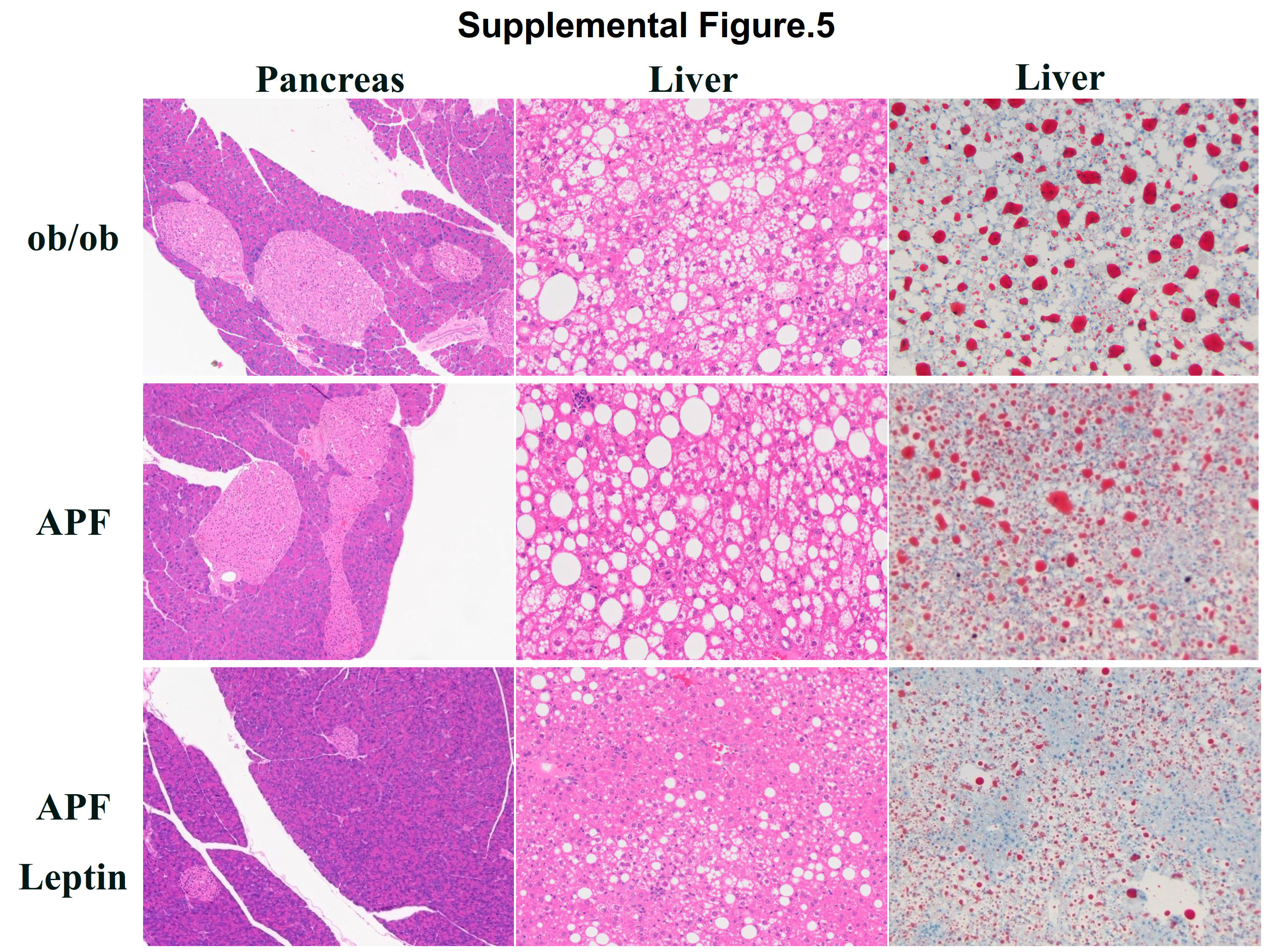

Supplement: Supplementary file 2 [file Image5.jpg]

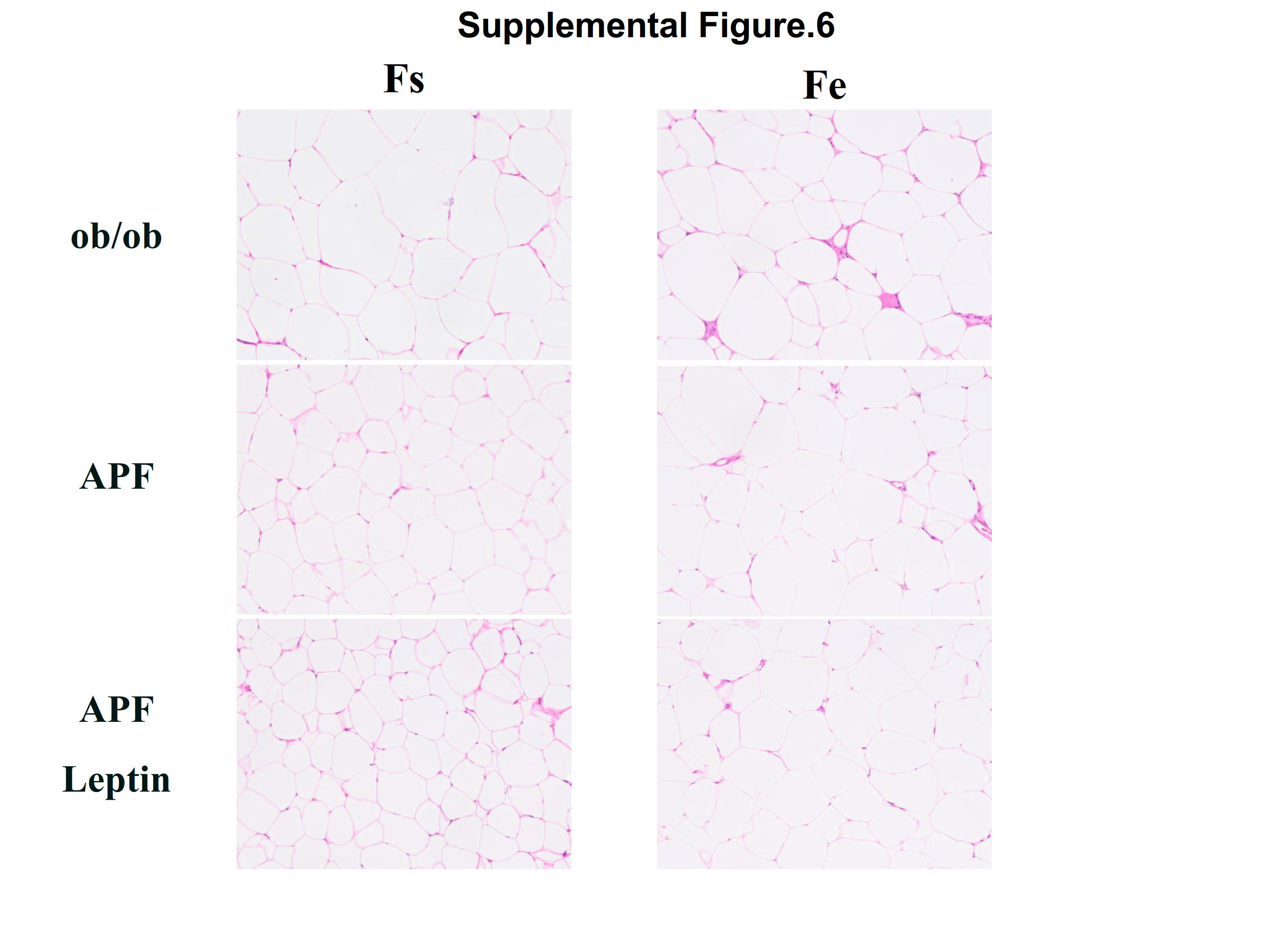

Supplement: Supplementary file 3 [file Image6.jpg]

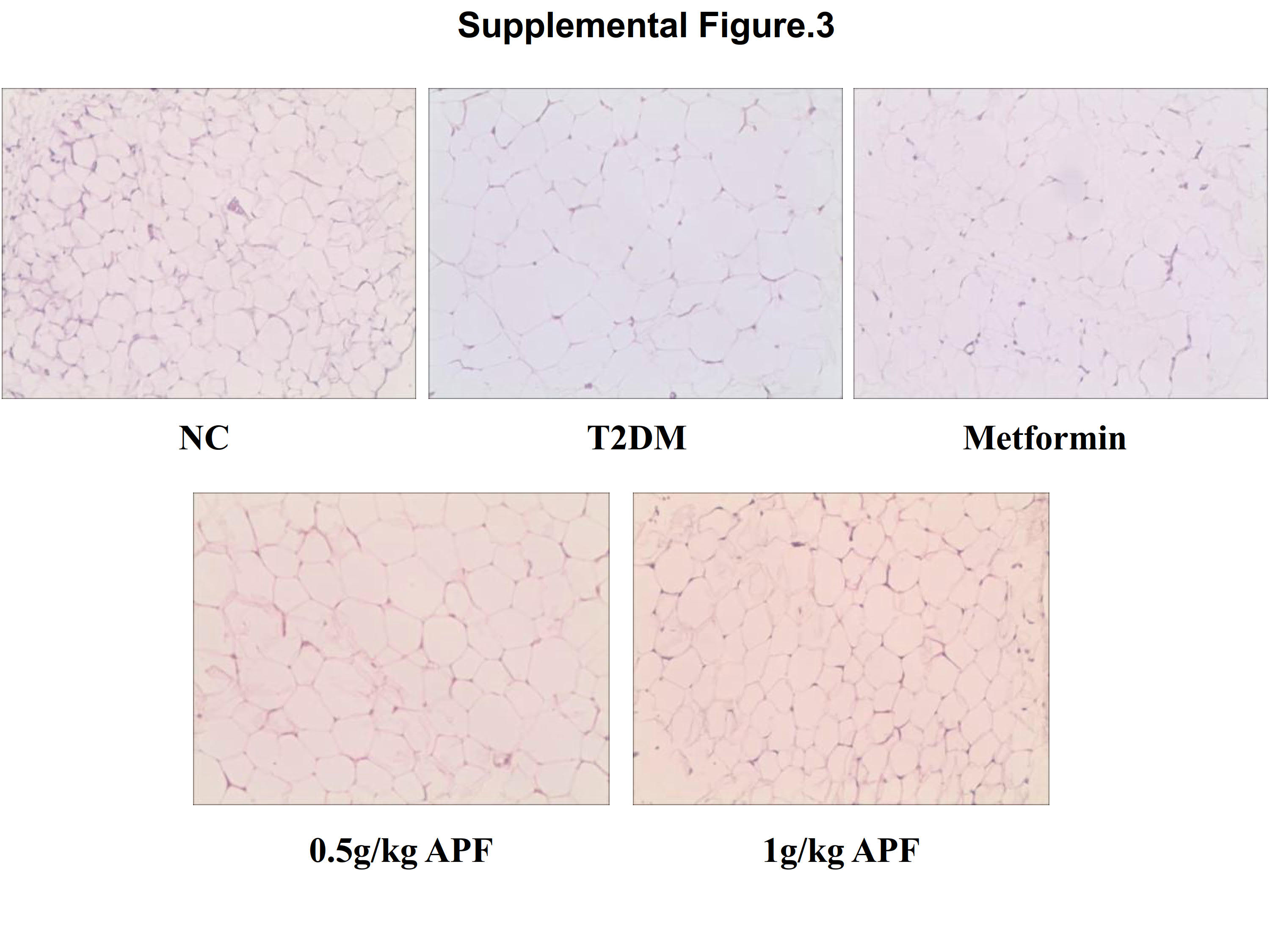

Supplement: Supplementary file 4 [file Image3.jpg]

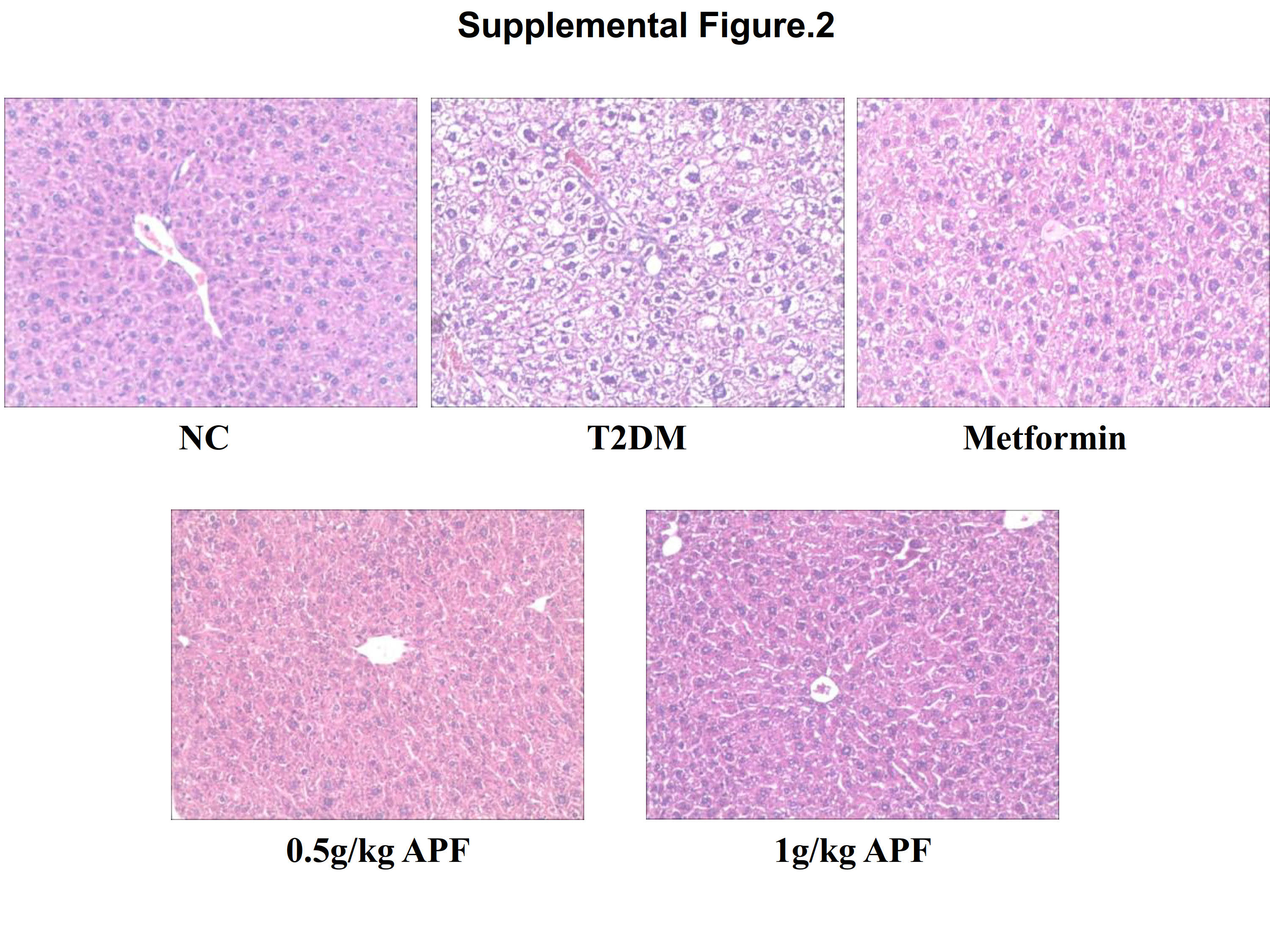

Supplement: Supplementary file 5 [file Image2.jpg]

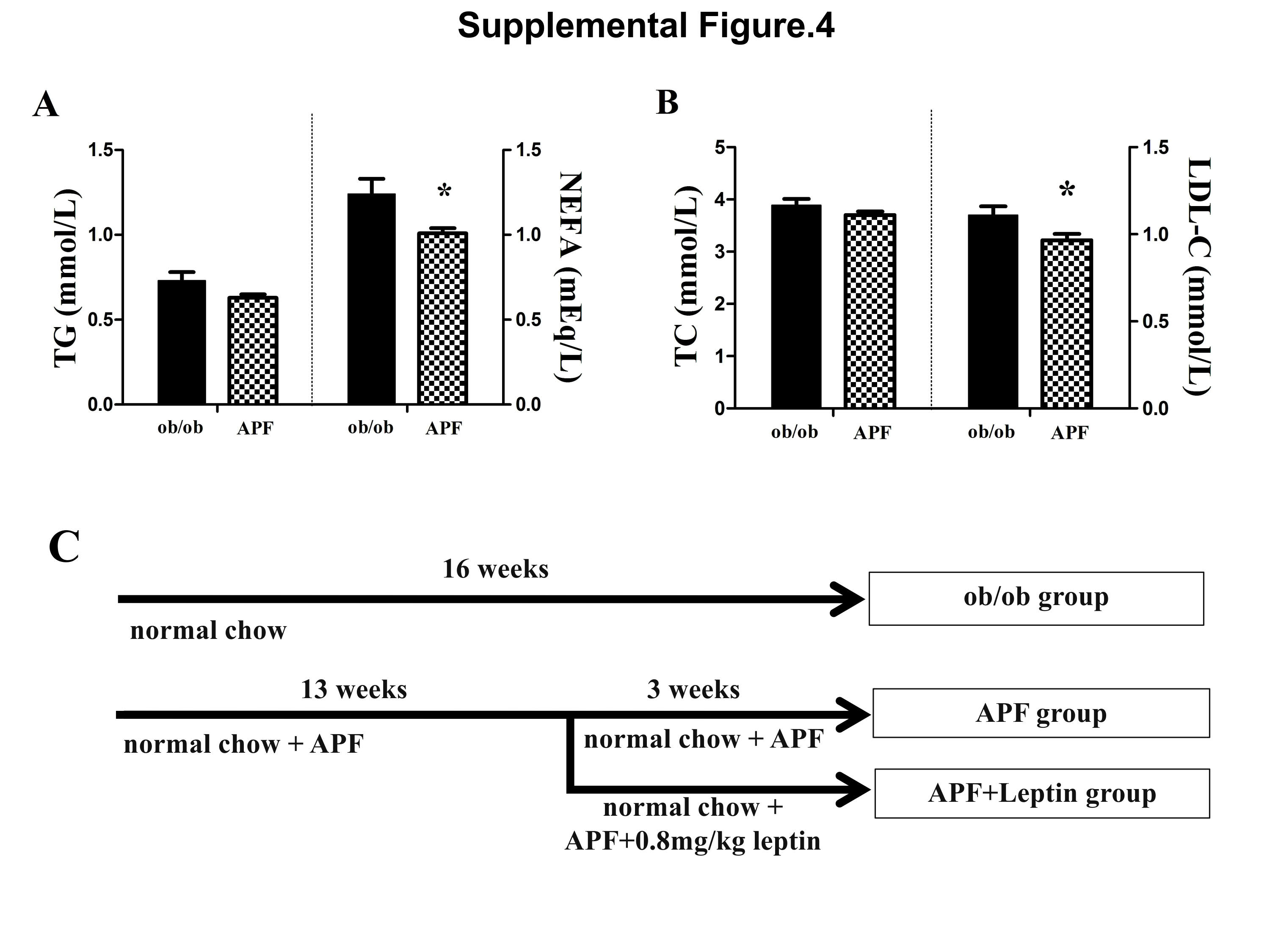

Supplement: Supplementary file 7 [file Image4.jpg]

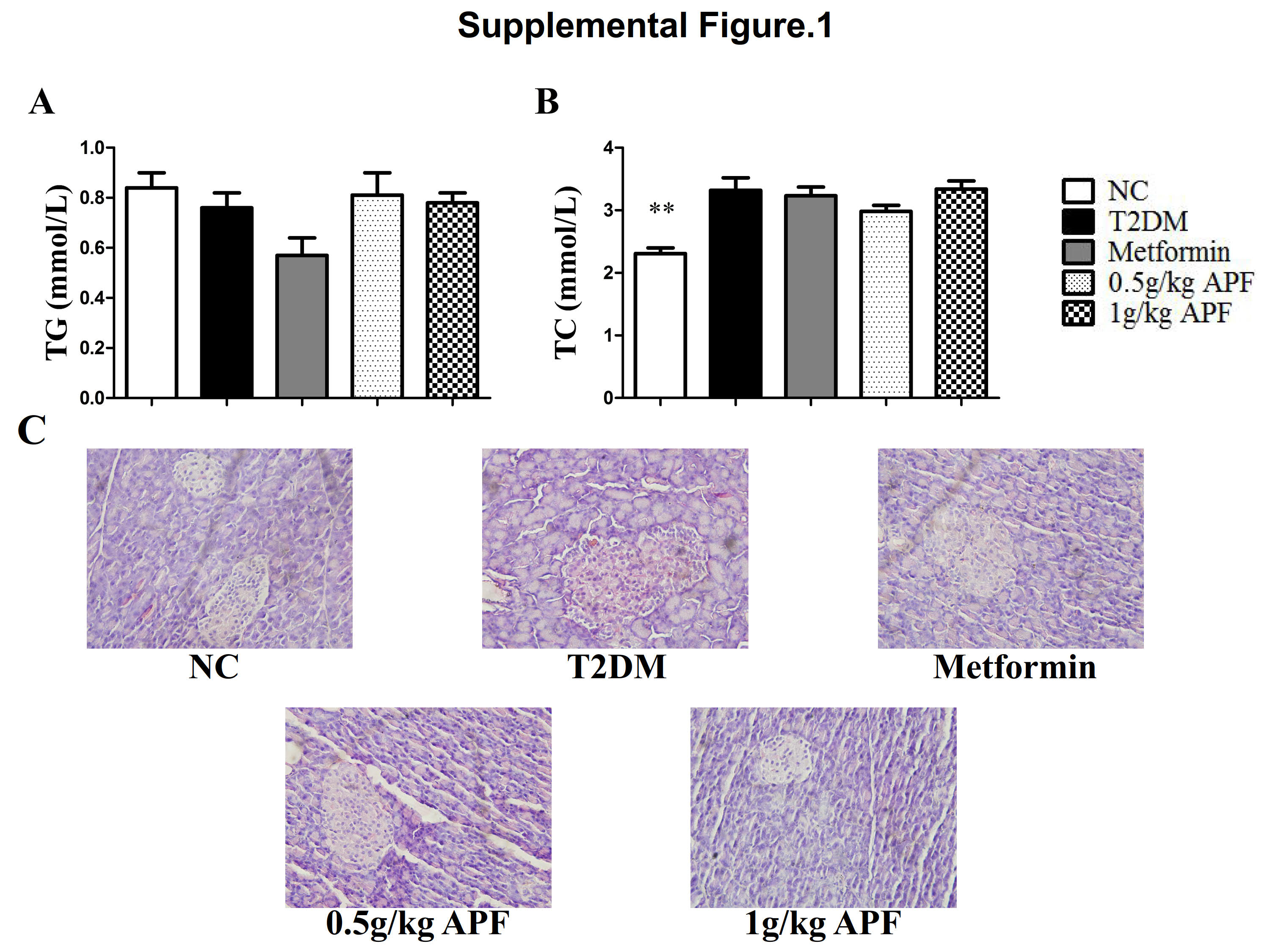

Supplement: Supplementary file 8 [file Image1.jpg]
