# Supplementary material for: Astragalus mongholicus powder, a traditional Chinese medicine formula ameliorate type 2 diabetes by regulating adipoinsular axis in diabetic mice
Source: Front Pharmacol. 2022 Aug 15;13:973927. doi: 10.3389/fphar.2022.973927 (PMC9420938; doi:10.3389/fphar.2022.973927)
Supplement: Supplementary file 6 [file DataSheet1.pdf]

Supplementary data

1. Determination of pueparin and calycosin-o-β-D-glucopyranoside

Chromatographic Column: luna 5u C18 (2) (250mm×4.60mm, 5um).

Mobile phase: methanol-water.

Flow velocity: 1.0 mL·min^{-1}.

Detection wavelength: 252 nm.

Evaporating temperature: 27 °C.

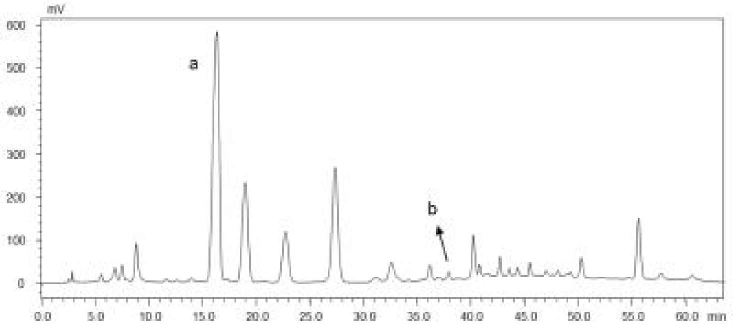

Fig. 1. The HPLC-ELSD chromatogram of pueparin (a) and calycosin-o- β -D-glucopyranoside (b)

Table1 Gradient elution mode of mobile phase

Time/min	Methanol (%)	Water (%)
0-25	25	75
25-45	25→45	75→55
45-55	45	55
55-60	45→25	55→75
60-70	25	75

2. Determination of Astragaloside IV

Chromatographic Column: JADE-PAK®DS-AQ (C18) (250mm×4.60mm, 5um).

Mobile phase: acetonitrile-water (35:65).

Flow velocity: 1.0 mL·min^{-1}.

Detection wavelength: 265 nm.

Evaporating temperature: 30 °C.

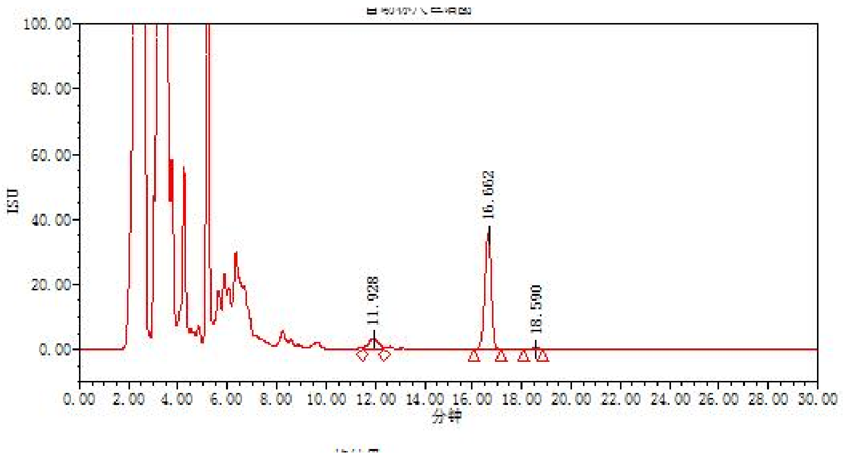

Fig. 2. The HPLC-ELSD chromatogram of Astragaloside IV.
